# Association between type of anesthesia and length of hospital stay in primary unilateral total knee arthroplasty patients: a single-center retrospective study

**DOI:** 10.1186/s13018-021-02817-4

**Published:** 2021-11-15

**Authors:** Xiaoqing Wang, He Li, Conghu Yuan, Hang Zhao

**Affiliations:** 1 Department of Anesthesiology, Affiliated Yancheng Hospital, School of Medicine, Southeast University, Juchang Road, Yandu District, Yancheng City, Jiangsu Province China; 2 Department of Anesthesiology, Affiliated Shuguang Hospital, Shanghai University of Traditional Chinese Medicine, Shanghai, China

**Keywords:** Type of anesthesia, Length of hospital stay, Total knee arthroplasty, Retrospective study

## Abstract

**Objective:**

This study explored the risk factors influencing the length of hospital stay (LOS) and establish whether the type of anesthesia is independently associated with the LOS in patients after primary unilateral total knee arthroplasty (TKA).

**Methods:**

In this retrospective cohort study, 2309 patients undergoing unilateral TKA were recruited between January 2013 and June 2014 in a tertiary academic medical center in Singapore. Univariate and multivariate linear regression analyses were used to identify the independent risk factors associated with LOS. Besides, subgroup and interaction analyses were performed to evaluate the relationship between the type of anesthesia and LOS.

**Result:**

In total, 2309 patients were identified. Out of these, 791 patients underwent general anesthesia, whereas 1518 patients underwent regional anesthesia. Multivariate regression analyses revealed that prolonged LOS was significantly associated with age ≥ 65 years (*β* = 0.48; 95% CI, 0.09–0.87; *P* = 0.015), diabetes mellitus (DM) (*β* = 0.8; 95% CI, 0.33–1.27; *P* = 0.001), congestive cardiac failure (CCF) (*β* = 4.1; 95% CI, 2.02–6.17; *P* < 0.001), perioperative blood transfusion (*β* = 5.71; 95% CI, 4.86–6.56; *P* < 0.001), creatinine > 2 mg/dL (*β* = 4.54; 95% CI, 2.46–6.62; *P* < 0.001), ASA status (III) (*β* = 1.72; 95% CI, 0.72–2.71; *P* = 0.001), general anesthesia (*β* = 0.78; 95% CI, 0.41–1.66; *P* < 0.001). The LOS further decreased among participants receiving regional anesthesia at advanced age (age ≥ 65 years) (*β* = − 1.12; 95% CI, − 1.66 to − 0.58; *P* < 0.001), patients with BMI ≤ 25 kg/m^2^ (*β* = − 1.92; 95% CI, − 2.73 to − 1.11; *P* < 0.001) or ≥ 30 kg/m^2^ (*β* = − 0.58; 95% CI, − 1.1 to − 0.06; *P* = 0.029).

**Conclusion:**

Our findings demonstrated that age ≥ 65 years, DM, CCF, perioperative blood transfusion, creatinine > 2 mg/dL, ASA status (III), general anesthesia are associated with a prolonged LOS after primary TKA. Elderly patients (age ≥ 65 years) and patients with BMI ≤ 25 kg/m^2^ or ≥ 30 kg/m^2^ receiving regional anesthesia have a further reduced LOS. Therefore, when TKA is performed, priority for regional anesthesia is given to the elderly patients (age ≥ 65 years old) and those with BMI ≤ 25 kg/m^2^ or ≥ 30 kg/m^2^.

**Supplementary Information:**

The online version contains supplementary material available at 10.1186/s13018-021-02817-4.

## Introduction

With the aging population, there has been a significant increase in the number of people suffering from arthritis. The World Health Organization (WHO) has globally estimated that over 250 million individuals are affected by arthritis, causing significant disability and reduced quality of life [1]. Knee osteoarthritis (KOA) is the most prevalent degenerative disorder of joints with chronic pain and mobility restraint [2]. Notably, total knee arthroplasty (TKA) is the most effective surgical procedure for end-stage osteoarthritis [3, 4]. In 2018, over 1.3 million TKA cases were performed in the United States, this number is annually increasing [5]. Meanwhile, the cases of TKA increased from 53,880 to 374,833 in mainland China within the recent 10 years, i.e., a 5.9-fold increase [6]. An increasing incidence of TKA poses a significant economic burden to the healthcare system. For instance, US medicare expenditure has statistically continued to increase and currently accounts for approximately 18% of the economy [7]. Thus, the health care system is struggling to guarantee quality health care and reduce hospital costs [8, 9]. Length of hospital stay (LOS) has a significant effect on overall healthcare expenditure and is considered a clinical proxy for the value of care. Generally, LOS is considered a critical measurement of healthcare efficiency and resource utilization [10]. Reduced LOS minimizes the infection risk and medication side effects [11]. It improves the treatment quality and increases hospital profit with efficient bed management [12]. LOS may be influenced by several factors, including patient characteristics (age, gender, BMI, comorbidities, the American Society of Anesthesiologists (ASA) classification); perioperative management (anesthesia type, blood management) [13], surgical characteristics (approach, prosthesis design,duration time) [14–16], postoperative management (mobilization timing, postoperative pain) [17–21], and postoperative complication before discharge (infection, deep vein thrombosis) [11].

Anesthesia is an important step to ensure the success of the surgery. Therefore, the selection of an anesthetic method is of utmost importance for the outcomes of patients undergoing TKA. General and/or regional anesthesia is appropriate for TKA and is familiar to most anesthesiologists. Anesthesiologists typically select the type of anesthesia based on their practice style and various patient-related factors. General anesthesia is associated with higher rates of postoperative nausea, vomiting, and delirium. On the other hand, regional anesthesia may be complicated by block failure and destructive complications including epidural abscess, spinal hematoma, and nerve injury [22]. The complications above prolong the LOS. However, an anesthetic method that causes reduced LOS in TKA patients is not known.

Previous studies investigating the relationship between anesthetic technique and outcomes revealed that patients undergoing TKA under regional anesthesia had reduced LOS compared to those undergoing general anesthesia [23, 24]. Nonetheless, recent high‐quality randomized controlled trials on the relationship between anesthetic technique and outcomes in TKA patients have yielded conflicting results [25, 26]. This study seeks to identify the risk factors influencing LOS and examine the relationship of anesthetic technique with LOS among patients undergoing TKA in a tertiary academic medical center in Singapore between January 2013 and June 2014. We speculated that regional anesthesia is associated with reduced LOS compared to general anesthesia.

## Materials and methods

### Data sources

This retrospective population study was conducted using data from the Dryad Digital Repository (https://datadryad.org/stash/dataset/doi:10.5061/dryad.73250). This website permitted users to freely download the raw data. Authors of the original study have authorized the ownership of the original data to the data-dryad Web site. According to Dryad Terms of Service, we cited Dryad data Package for secondary analysis on a different hypothesis without infringing on the authors’ rights**.**

### Study population

**Fig. 1:**
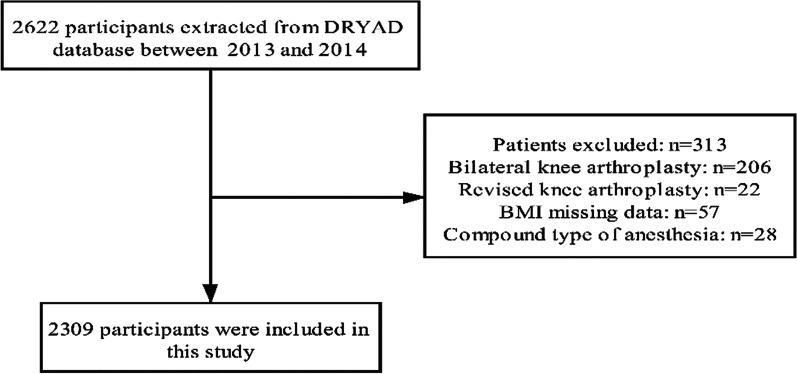
Flowchart detailing the selection process for patients included in this retrospective analysis

We retrospectively analyzed data of 2622 patients, who underwent TKA between January 2013 and June 2014 in a tertiary academic medical centre in Singapore [27]. These clinical records were extracted from the institution's clinical information system (Sunrise Clinical Manager (SCM), Allscripts, Illinois, USA) and stored in their enterprise data repository and analytics system (SingHealth-IHiS Electronic Health Intelligence System), which integrates information from administration, clinical and ancillary healthcare systems. Additionally, Institutional Review Board approval was obtained (Sing-health CIRB 2014/651/D) prior to the start of the study. In the present study, it was performed to address the relationship between the type of anesthesia and LOS. The target independent variable is type of anesthesia (regional anesthesia and general anesthesia) and the dependent variable was LOS obtained at baseline. The LOS was defined as time from the date of hospital admission to the date of discharge. After excluding 22 patients who underwent revised TKA and 206 patients who underwent bilateral TKA, 57 patients with missing BMI, 28 patients who underwent a compound type of anesthesia. A total of 2309 patients underwent primary unilateral TKA were recruited for the final analysis (Fig. 1).

### Measurement of covariants

Data was obtained the clinical information from the Dryad Digital Repository. Covariates included in this study were treated as potential confounding factors on the relationship of type of anesthesia and LOS in TKA patients based on previous studies. The major covariates of this study included patient demographics such as age, sex, race, body mass index (BMI); preoperative comorbidities such as smoking, preoperative hemoglobin (Hb) level,American Society of Anesthesiologist Physical Status (ASA-PS) score, individual components of the Revised Risk Cardiac Index (RCRI), such as a history of previous cerebrovascular accidents (CVAs), ischaemic heart disease (IHD), congestive cardiac failure (CCF), diabetes mellitus (DM) and elevated preoperative creatinine level > 2 mg/dL; details of the operation such as duration of operation, perioperative blood transfusion. American Society of Anesthesiologists-Physical Status (ASA-PS) follows that of the ASA-PS definitions. Perioperative blood transfusion was defined as within 2 weeks prior to surgery to 2 weeks following surgery. By convention, antiplatelet medications (except aspirin) were discontinued for the recommended duration before the procedure. the use of intravenous tranexamic acid and intraarticular tranexamic acid intra-operatively and drainage tube implantation into the joint post-operatively were not standardized. All patients received standard postoperative care of TKA protocol. This included subcutaneous administration of 40 mg low molecular weight heparin (Clexane, Sanofi, Paris, France) once daily for thromboembolism prophylaxis on the first postoperative day until discharge. Patients who met the following specified criteria established by the surgeon and physiotherapist were discharged from the hospital: owning the ability to flex the operated knee close to 90°, turn around with the assistance of a walking frame and climb up a few steps.

### Statistical analysis

The distribution of continuous variables was described with the use of the mean and the standard deviation (SD) or the median and the interquartile range [IQR]. Categorical variables were presented as proportions and percentages of the total. For confounders with partial missing data, if it is a categorical variable, we directly treated it as a new independent group; if it is a continuous variable, we replaced it with an average or median value. The differences between groups were checked by *x*^2^ test or Fisher’s Exact test for categorical variables or by the student’s t-test or Mann–Whitney *U* test for continuous variables. Multivariate linear regression models were utilized to calculate regression coefficients *β* and 95% confidence interval (CI) for estimating the association between type of anesthesia and LOS. We estimated three models: Model 1 did not adjust any confounders. Model 2 adjusted for age, sex, and race. Model 3 additionally adjusted for age, sex, race, BMI, smoking, preoperative Hb level, DM, perioperative blood transfusion, Creatinine > 2 mg/dL, previous IHD, CCF, CVA, ASA-PS, duration of operation. Interaction and stratified analyses were conducted according to age (< 65 and ≥ 65 years), gender (male and female), BMI (< 25, 25–29.9 and ≥ 30 kg/m^2^), Hb(< 11, 11–12.9 and ≥ 13 g/dL), ASA status (I, II and III), operation duration (< 60, 60–89 and ≥ 90 min), DM (diabetes and non-diabetes). Subgroup analyses were performed using stratified linear regression models. Account for non-linear relationship between age or BMI and LOS by category of anesthetic methods, we also used Generalized additive model and the smooth curve fitting (penalized spline method) to address nonlinearity. *P* < 0.05 was considered statistically significant. This study adheres to the Strengthening the Reporting of Observational Studies in Epidemiology guidelines. All the analyses were performed with the statistical soſtware packages R 3.3.2 (http://www.R-project.org, The R Foundation) and Free Statistics software versions 1.1.

## Results

### Baseline characteristics of study subjects

We identified 2622 patients underwent TKA between January 2013 and June 2014 in a tertiary academic medical centre in Singapore. After applying the exclusion criteria, the analysis sample included 2309 eligible patients (1746 female and 563 male). The characteristics of the subjects between the general anesthesia and regional anesthesia group are presented in Table 1. Among these, 791 (34.3%) individuals received general anesthesia, 1518 (65.7%) individuals received regional anesthesia. Of the total participants, 84.2% (*n* = 1945) were Chinese, 6.9% (*n* = 159) Malay, 5.9% (*n* = 137) Indian, and 2.9% (*n* = 68) others. The mean age of the sample was 66.5 years old (SD 8.1). The median LOS was 4.0 days (IQR 3.0–6.0). The mean BMI was 27.8 kg/m^2^ (SD 5.7). In the overall study population, patients who received regional anesthesia were older (*P* < 0.001, ►Table 1), more likely to be female (*P* = 0.003, ►Table 1) and to have shorter LOS (*P* < 0.001, ►Table 1), and tended not to require blood transfusion treatment (*P* = 0.021, ►Table 1).

**Table 1:** Baseline characteristics of patients

Variables	Total (*n* = 2309)	GA (*n* = 791)	RA (*n* = 1518)	*P* values
*Patient demographics*				
Race, *n* (%)				0.019
Chinese	1945 (84.2)	642 (81.2)	1303 (85.8)	
Malay	159 (6.9)	63 (8)	96 (6.3)	
Indian	137 (5.9)	61 (7.7)	76 (5)	
Others	68 (2.9)	25 (3.2)	43 (2.8)	
Gender, *n* (%)				0.003
Female	1746 (75.6)	628 (79.4)	1118 (73.6)	
Male	563 (24.4)	163 (20.6)	400 (26.4)	
BMI (Kg/m^2^)	27.8 ± 5.7	28.0 ± 4.7	27.7 ± 6.2	0.338
Age(years)	66.5 ± 8.1	64.7 ± 8.3	67.4 ± 7.9	< 0.001
ASA status, *n* (%)				0.009
I	155 (6.7)	68 (8.6)	87 (5.7)	
II	2016 (87.3)	668 (84.5)	1348 (88.8)	
III	138 (6.0)	55 (7)	83 (5.5)	
Details of operation				
LOS(Days)	4.0 (3.0, 6.0)	4.0 (4.0, 6.0)	4.0 (3.0, 5.0)	< 0.001
Operation duration (mins)	75.0 (65.0, 95.0)	80.0 (65.0, 95.0)	75.0 (65.0, 95.0)	0.228
Day of week of operation, *n* (%)				0.142
Monday	381 (16.5)	144 (18.2)	237 (15.6)	
Tuesday	526 (22.8)	184 (23.3)	342 (22.5)	
Wednesday	395 (17.1)	126 (15.9)	269 (17.7)	
Thursday	522 (22.6)	161 (20.4)	361 (23.8)	
Friday	365 (15.8)	127 (16.1)	238 (15.7)	
Saturday	120 (5.2)	49 (6.2)	71 (4.7)	
Perioperative blood transfusion, *n* (%)				0.021
No	2200 (95.3)	742 (93.8)	1458 (96)	
Yes	109 (4.7)	49 (6.2)	60 (4)	
Patient comorbidities				
Preoperative Hb (g/dL)	13.1 ± 1.5	13.1 ± 1.5	13.1 ± 1.4	0.57
Smoking, *n* (%)				0.069
No	2088 (90.4)	728 (92)	1360 (89.6)	
Yes	221 (9.6)	63 (8)	158 (10.4)	
OSA, *n* (%)				0.672
No	2098 (90.9)	722 (91.3)	1376 (90.6)	
Yes	211 (9.1)	69 (8.7)	142 (9.4)	
DM, *n* (%)				1.000
No	1869 (80.9)	640 (80.9)	1229 (81)	
Yes	440 (19.1)	151 (19.1)	289 (19)	
IHD, *n* (%)				0.950
No	2184 (94.6)	749 (94.7)	1435 (94.5)	
Yes	125 (5.4)	42 (5.3)	83 (5.5)	
CCF, *n* (%)				0.088
No	2292 (99.3)	789 (99.7)	1503 (99)	
Yes	17 (0.7)	2 (0.3)	15 (1)	
CVA, *n* (%)				1.000
No	2266 (98.1)	776 (98.1)	1490 (98.2)	
Yes	43 (1.9)	15 (1.9)	28 (1.8)	
Creatinine > 2 mg/dL, *n* (%)				0.719
No	2041 (88.4)	702 (88.7)	1339 (88.2)	
Yes	17 (0.7)	7 (0.9)	10 (0.7)	
NA	251 (10.9)	82 (10.4)	169 (11.1)	

Data presented are mean ± SD, median (Q1–Q3), or *n* (%)

*GA* general anesthesia, *RA* regional anesthesia, *BMI* body mass index, *ASA Status* American Society of Anesthesiologist Physical Status, *Hb* hemoglobin, *LOS* length of stay, *OSA* Obstructive sleep apnea, *DM* diabetes mellitus, *IHD* ischemic heart disease, *CCF* congestive cardiac failure, *CVA* cerebrovascular accidents, *SD* standard deviation, *NA* not recorded

### Risk factors and length of hospital stay

The results of univariate analysis are presented in Table 2. The risk factors of prolonged LOS include that age ≥ 65 years old (*β* = 0.77; 95% CI, 0.39–1.15; *P* < 0.001), operation duration ≥ 90 min (*β* = 0.66; 95% CI, 0.05–1.28; *P* = 0.03), general anesthesia (*β* = 0.84; 95% CI, 0.44–1.23; *P* < 0.001), perioperative blood transfusion (*β* = 6.81; 95% CI, 5.96–7.65; *P* < 0.001), ASA(III) (*β* = 2.95; 95% CI, 1.9–4; *P* < 0.001), DM (*β* = 1.03; 95% CI, 0.55–1.51; *P* < 0.001), IHD (*β* = 1.37; 95% CI, 0.54–2.2; *P* = 0.001), CCF (*β* = 6.5; 95% CI, 4.30–8.69; *P* < 0.001), creatinine > 2 mg/dL (*β* = 6.86; 95% CI, 4.67–9.06; *P* < 0.001). In addition, multivariable logistic regression analyses are conducted for the independent effects of age, operation duration, type of anethesia, perioperative blood transfusion, ASA status, DM, IHD, CCF, creatinine > 2 mg/dL in Table 3. The result reveals that age ≥ 65 years old (*β* = 0.48; 95% CI, 0.09–0.87; *P* = 0.015), DM (*β* = 0.8; 95% CI, 0.33–1.27; *P* = 0.001), CCF (*β* = 4.1; 95% CI, 2.02–6.17; *P* < 0.001), perioperative blood transfusion (*β* = 5.71; 95% CI, 4.86–6.56; *P* < 0.001), creatinine > 2 mg/dL (*β* = 4.54; 95% CI, 2.46–6.62; *P* < 0.001), ASA status(III) (*β* = 1.72; 95% CI, 0.72–2.71; *P* = 0.001), general anesthesia(*β* = 0.78; 95% CI, 0.41–1.66; *P* < 0.001) was significantly associated with prolonged LOS after adjusting for other covariates. So that LOS was 0.78 days longer in general anesthesia group compared with regional anesthesia group. In analysis, we further explored the association between type of anethesia and LOS. As shown in Table 4, the stratified analysis revealed a highly consistent pattern. Regardless of subgroup, effect size of type of anethesia on LOS were stable. The interaction analysis revealed that age (*P* for interaction = 0.006) and BMI (*P* for interaction = 0.01) played an interactive role in the association between type of anethesia and LOS (Table 4). The participants aged ≥ 65 years old (*β* = −1.12; 95% CI, −1.66 to − 0.58; *P* < 0.001) had shorter LOS in regional anesthesia group than those aged < 65 years old (*β* = − 0.27; 95% CI, − 0.73 to 0.19; *P* = 0.247). The association between age and LOS in different anesthesia group is presented in Addtional file 1. In addition, participants with BMI < 25 kg/m^2^ (*β* = − 1.92; 95% CI, − 2.73 to − 1.11; *P* < 0.001) or ≥ 30 kg/m^2^ (*β* = − 0.58; 95% CI, − 1.1 to − 0.06; *P* = 0.029) had a shorter LOS in regional anesthesia group in than BMI at 25–30 kg/m^2^ (*β* = − 0.31; 95% CI, − 0.92 to 0.29; *P* = 0.313). The association between BMI and LOS in different anesthesia group is presented in Addtional file 2.

**Table 2:** Univariate analysis for LOS

Covariate	*β* (95%CI)	*P* value
*Patient demographics*		
Race		
Chinese	Reference	
Malay	− 0.44 (− 1.19, 0.31)	0.248
Indian	0.12 (− 0.68, 0.92)	0.766
Others	0.51 (− 0.61, 1.63)	0.368
Gender		
Female	Reference	
Male	− 0.1 (− 0.54, 0.34)	0.669
Age(years)		
< 65	Reference	
≥ 65	0.77 (0.39, 1.15)	< 0.001
BMI (kg/m^2^)		
< 25	Reference	
25–29.9	− 0.29 (− 0.74, 0.16)	0.205
≥ 30	0.26 (− 0.76, 0.23)	0.299
*Details of operation*		
Type of Anaesthesia		
GA	0.84 (0.44, 1.23)	< 0.001
RA	Reference	
Operation duration (mins)		
< 60	Reference	
60–90	0.16 (− 0.42, 0.73)	0.594
≥ 90	0.66 (0.05, 1.28)	0.033
Day of week of operation		
Monday	1.26 (0.66,1.87)	< 0.001
Tuesday	1.00 (0.44, 1.56)	< 0.001
Wednesday	0.74 (0.14, 1.34)	0.016
Thursday	Reference	
Friday	1.5 (0.88, 2.11)	< 0.001
Saturday	0.11 (− 0.81, 1.02)	0.82
Perioperative blood transfusion		
No	Reference	
Yes	6.81 (5.96, 7.65)	< 0.001
*Patient comorbidities*		
ASA status		
I	Reference	
II	0.37 (− 0.38, 1.12)	0.339
III	2.95 (1.9, 4)	< 0.001
Preoperative Hb (g/dL)		
< 11	1.28 (0.51, 2.05)	0.001
11–12.9	0.63(0.24, 1.03)	0.002
≥ 13	Reference	
Smoking		
No	Reference	
Yes	− 0.37 (− 1.02, 0.27)	0.253
OSA		
No	Reference	
Yes	− 0.51 (− 1.16, 0.15)	0.129
DM		
No	Reference	
Yes	1.03 (0.55, 1.51)	< 0.001
IHD		
No	Reference	
Yes	1.37 (0.54, 2.2)	0.001
CCF		
No	Reference	
Yes	6.5 (4.30, 8.69)	< 0.001
CVA		
No	Reference	
Yes	1.04 (− 0.36, 2.44)	0.144
Creatinine > 2 mg/dL		
No	Reference	
Yes	6.86 (4.67, 9.06)	< 0.001
NA	0.09 (− 0.51, 0.69)	0.770

*GA* general anesthesia, *RA* regional anesthesia, *BMI* body mass index, *ASA Status* American Society of Anesthesiologist Physical Status, *Hb* haemoglobin, *LOS* length of stay, *OSA* Obstructive sleep apnea, *DM* diabetes mellitus, *IHD* ischemic heart disease, *CCF* congestive cardiac failure, *CVA* cerebrovascular accidents, *SD* standard deviation, *NA* not recorded

**Table 3:** Multivariate analyses of risk factors associated with LOS

	Model I		Model II		Model III	
	*β* (95%CI)	*P* value	*β* (95%CI)	*P* value	*β* (95%CI)	*P* value
Age (years)						
Per 10	0.68 (0.45–0.91)	< 0.001	0.69 (0.46–0.93)	< 0.001	0.5 (0.27–0.73)	< 0.001
< 65	Reference		Reference		Reference	
≥ 65	0.77 (0.39–1.15)	< 0.001	0.77 (0.38–1.16)	< 0.001	0.48 (0.09–0.87)	0.015
Hb (g/dL)						
< 11	Reference		Reference		Reference	
11–12.9	− 0.65 (− 1.43 to 0.14)	0.109	− 0.62 (− 1.4 to 0.17)	0.124	0.34 (− 0.41 to 1.08)	0.374
≥ 13	-1.28 (-2.05 to − 0.51)	0.001	− 1.12 (− 1.89 to − 0.35)	0.004	0.13 (− 0.6 to 0.87)	0.721
DM						
No	Reference		Reference		Reference	
Yes	1.03 (0.55–1.51)	< 0.001	0.97 (0.49–1.45)	< 0.001	0.8 (0.33–1.27)	0.001
CCF						
No	Reference		Reference		Reference	
Yes	6.5 (4.3–8.69)	< 0.001	6.28 (4.1–8.47)	< 0.001	4.1 (2.02–6.17)	< 0.001
IHD						
No						
Yes						
Operation duration (mins)						
< 60	Reference		Reference		Reference	
60–90	0.16 (− 0.42 to 0.73)	0.594	0.19 (− 0.38 to 0.77)	0.513	0.17 (− 0.37 to 0.7)	0.544
≥ 90	0.66 (0.05–1.28)	0.033	0.72 (0.11–1.32)	0.021	0.49 (− 0.09 to 1.07)	0.095
Perioperative blood transfusion						
No	Reference		Reference		Reference	
Yes	6.81 (5.96–7.65)	< 0.001	6.62 (5.77–7.47)	< 0.001	5.71 (4.86–6.56)	< 0.001
Creatinine > 2 mg/dL						
No	Reference		Reference		Reference	
Yes	6.86 (4.67–9.05)	< 0.001	6.89 (4.7–9.07)	< 0.001	4.54 (2.46–6.62)	< 0.001
NA	0.09 (− 0.51 to 0.69)	0.77	0.11 (− 0.49 to 0.71)	0.722	0.09 (− 0.47 to 0.66)	0.744
ASA status						
I	Reference		Reference		Reference	
II	0.37 (-0.38–1.12)	0.339	0.2 (− 0.55 to 0.95)	0.596	0.15 (− 0.56 to 0.85)	0.684
III	2.95 (1.9–4)	< 0.001	2.68 (1.62–3.73)	< 0.001	1.72 (0.72–2.71)	0.001
Type of anesthesia						
GA	0.84 (0.44–1.23)	< 0.001	1.03 (0.63–1.43)	< 0.001	0.78 (0.41–1.66)	< 0.001
RA	Reference		Reference		Reference	

Data presented are *β* and 95% CIs

Model I: We did not adjust any covariants

Model II: We adjusted age, gender and race

Model III: We adjusted age, gender, race, ASA status, Hb, smoking, Operation Duration, BMI, DM, creatinine > 2 mg/dL, IHD, CCF, CVA, Type of anesthesia, Day of week of operation and the perioperative blood transfusion. In each case, the model is not adjusted for the variable itself

*GA* general anesthesia, *RA* regional anesthesia, *ASA Status* American Society of Anesthesiologist Physical Status, *Hb* hemoglobin, *DM* diabetes mellitus, *IHD* ischemic heart disease, *CCF* congestive cardiac failure

**Table 4:** Effect size of type of anesthesia on LOS in each group

Subgroup	*N*	*β* (95% CI)	*P* value	*P* for interaction
Age(years)				0.006
< 65	940	− 0.27 (− 0.73 to 0.19)	0.247	
≥ 65	1369	− 1.12 (− 1.66 to − 0.58)	< 0.001	
Gender				0.063
Female	1746	− 0.68 (− 1.08 to − 0.29)	0.001	
Male	563	− 1.31 (− 2.30 to − 0.31)	0.010	
Preoperative Hb (g/dL)				0.934
< 11	156	− 1.49 (− 2.68 to − 0.3)	0.015	
11–12.9	868	− 0.78 (− 1.49 to − 0.07)	0.032	
≥ 13	1286	− 0.79 (− 1.25 to − 0.34)	0.001	
BMI (kg/m^2^)				0.01
< 25	683	− 1.92 (− 2.73 to − 1.11)	< 0.001	
25–29.9	986	− 0.31(− 0.92 to 0.29)	0.313	
≥ 30	640	− 0.58(− 1.1 to − 0.06)	0.029	
ASA status				0.526
I	155	− 0.03 (− 0.79 to 0.73)	0.934	
II	2016	− 0.85 (− 1.23 to − 0.48)	< 0.001	
III	138	− 1.12(− 4.65 to 2.41)	0.536	
Operation duration (mins)				0.817
< 60	308	− 1.1 (− 1.81 to − 0.39)	0.003	
60–89	1231	− 0.82 (− 1.38 to − 0.26)	0.004	
≥ 90	773	− 0.72 (− 1.37 to − 0.08)	0.028	
DM				0.061
No	1869	− 0.64 (− 1.02 to − 0.26)	0.001	
Yes	440	− 1.6 (− 2.69 to − 0.51)	0.004	

We adjusted age, gender, race, ASA status, Hb, smoking, Operation Duration, BMI, DM, creatinine > 2 mg/dL, IHD, CCF, CVA, Type of anesthesia, Day of week of operation and the perioperative blood transfusion. In each case, the model is not adjusted for the variable itself

*BMI* body mass index, *ASA Status* American Society of Anesthesiologist Physical Status, *Hb* haemoglobin, *DM* diabetes mellitus

## Discussion

We retrospectively uncovered high-risk factors for prolonged LOS and evaluated the relationship between the anesthetic method and LOS in patients after primary unilateral TKA between January 2013 and June 2014. Age ≥ 65 years, DM, CCF, ASA status (III), general anesthesia were significant and independent risks for prolonged LOS in patients after TKA. Moreover, regional anesthesia was associated with a shorter hospital LOS compared to general anesthesia in patients after primary unilateral TKA. Patients in the regional anesthesia group had a 0.78 days shorter LOS than those in the general anesthesia group. Moreover, subgroup and interaction analyses revealed that the relationship between regional anesthesia and a shorter hospital LOS is modified by age and BMI. A stronger relationship between anesthetic type and LOS was detected in elderly patients (age ≥ 65 years) and those whose BMI was ≤ 25 kg/m^2^ or ≥ 30 kg/m^2^.

Consistent with our research findings, Gulraj S et al. performed a retrospective analysis of prospectively collected observational data from the National Joint Registry (NJR) and found that LOS (*β* = − 0.47 days; 95% CI, − 0.49 to − 0.45; *P* < 0.001) was reduced with regional anesthesia compared to general anesthesia in patients undergoing TKA [24]. To our knowledge, so far, this study has the largest sample size (*n* = 426,104). In contrast with our study, given that BMI is frequently missing in the NJR, multivariable logistic regression analysis was performed for all patient and surgical factors excluding BMI. Besides, significant confounders were overlooked in covariates, including perioperative blood transfusion, and preoperative Hb level.

This work utilized an extended model approach to adjust the potential confounders and performed subgroup and interaction analyses to ensure a stable relationship between the anesthetic method and LOS. One meta-analysis confirmed that regional anesthesia had a reduced LOS compared to general anesthesia in TKA and total hip replacement (THR); this was in line with our findings. However, this meta-analysis of LOS enrolled only 1240 patients undergoing TKA and THA who were not separately grouped. Our study had a larger cohort including primary unilateral TKA (*n* = 2622). In contradiction with our study result, Jared et al. [28]. conducted a retrospective study of the TKA cohort (*n* = 183,080) using the American College of Surgeons National Surgical Quality Improvement Program (NSQIP) database between 2011 and 2016, describing a statistically significant but clinically irrelevant increase (OR = 0.050; 95% CI, 0.023–0.078; *P* < 0.001). Unlike this study, LOS among those treated with general anesthesia was 0.78 days longer than those treated with regional anesthesia (*β* = 0.78; 95% CI, 0.41–1.66; *P* < 0.001) after adjusting for other confounding factors and was statistically and clinically meaningful. Additionally, Harsten et al. [25]. discovered that general anesthesia resulted in shorter LOS compared to regional anesthesia among 124 patients with osteoarthritis undergoing TKA at the Department of Orthopaedic Surgery, Ha¨ssleholm Hospital, Sweden between September 2011 and June 2012. Elsewhere, Palanne et al. [26]. found that spinal and general anesthesia did not differ in LOS among patients (*n* = 413) undergoing TKA. Although the above studies are randomized controlled trials (RCTs), the sample size is small from a single center. Therefore, the generalizability of the findings is at stake.

This paper has notable strengths. First, we used a large sample size. Secondly, we evaluated the relationship between anesthetic type and hospital LOS; different multivariate logistic regression models were used to minimize bias by adjusting for other confounding factors. Furthermore, a subgroup analysis was performed to improve the robustness of the results. Consequently, age and BMI significantly affected the relationship between the type of anesthesia and LOS through interaction analysis.

Nevertheless, this study has compelling limitations. First, it is based on a retrospective analysis from a published database. Although we attempted to statistically correct the bias, we could not exclude unmeasured and/or residual confounding factors vital for the outcomes of hospital LOS, including the use of a different agent and its duration; intraoperative blood loss and postoperative care techniques; postoperative pain which potentially brings bias to the results. Secondly, as a cross-sectional study design, it has less power to infer the causal relationship between anesthetic type and LOS. Thus, additional prospective follow-up studies are necessary to verify these findings. Thirdly, most of the patients recruited were Chinese in Singapore; different races may cause different results. Therefore, our results cannot necessarily be generalized to people of other ethnicities in different countries. Notably, regional anesthesia included intrathecal anesthesia, nerve block anesthesia, and local anesthesia. Importantly, this study did not specify the regional anesthetic method that had been used. As such, further exploratory studies are essential to analyze a more suitable regional anesthesia for TKA from the perspective of shortening LOS in the next step.

## Conclusion

In conclusion, the use of regional anesthesia is independently and positively related to shorter LOS compared to general anesthesia in patients after unilateral TKA. This impact is prominent among elderly patients (age ≥ 65 years) and those with BMI ≤ 25 kg/m^2^ or ≥ 30 kg/m^2^. For anesthesiologists, it is essential to identify high-risk factors of prolonged LOS and select the most appropriate anesthetic type for patients with primary unilateral TKA. Herein, we recommend regional anesthesia as the primary anesthetic approach for primary unilateral TKA, specifically among the elderly patients (age ≥ 65 years) and those with BMI ≤ 25 kg/m^2^ or ≥ 30 kg/m^2^.

## Supplementary Information

**Additional file 1**. The association between age and LOS in different anesthesia group.

**Additional file 2**. The association between BMI and LOS in different anesthesia group.

## Data Availability

Data can be downloaded from the Dryad Digital Repository. Dryad data package: Abdullah HR, Sim E, Hao Y, Lin G, Liew GHC, Lamoureux EL, Tan MH (2017). Data from: Association between preoperative anemia with length of hospital stay among patients undergoing primary total knee arthroplasty in Singapore: a single-center retrospective study. Dryad Digital Reposi-tory. https://doi.org/10.5061/dryad.73250.

## Abbreviations

BMI: Body mass index
ASA status: American Society of Anesthesiologist Physical status
Hb: Hemoglobin
TKA: Total knee arthroplasty
LOS: Hospital length of stay
OSA: Obstructive sleep apnea
DM: Diabetes mellitus
IHD: Ischemic heart disease
CCF: Congestive cardiac failure
CVA: Cerebrovascular accidents
SD: Standard deviation
NA: Not recorded
CI: Confidence interval
GA: General anesthesia
RA: Regional anesthesia
