# Supplementary figures and images for: Association between type of anesthesia and length of hospital stay in primary unilateral total knee arthroplasty patients: a single-center retrospective study
Source: J Orthop Surg Res. 2021 Nov 15;16:671. doi: 10.1186/s13018-021-02817-4 (PMC8591843; doi:10.1186/s13018-021-02817-4)

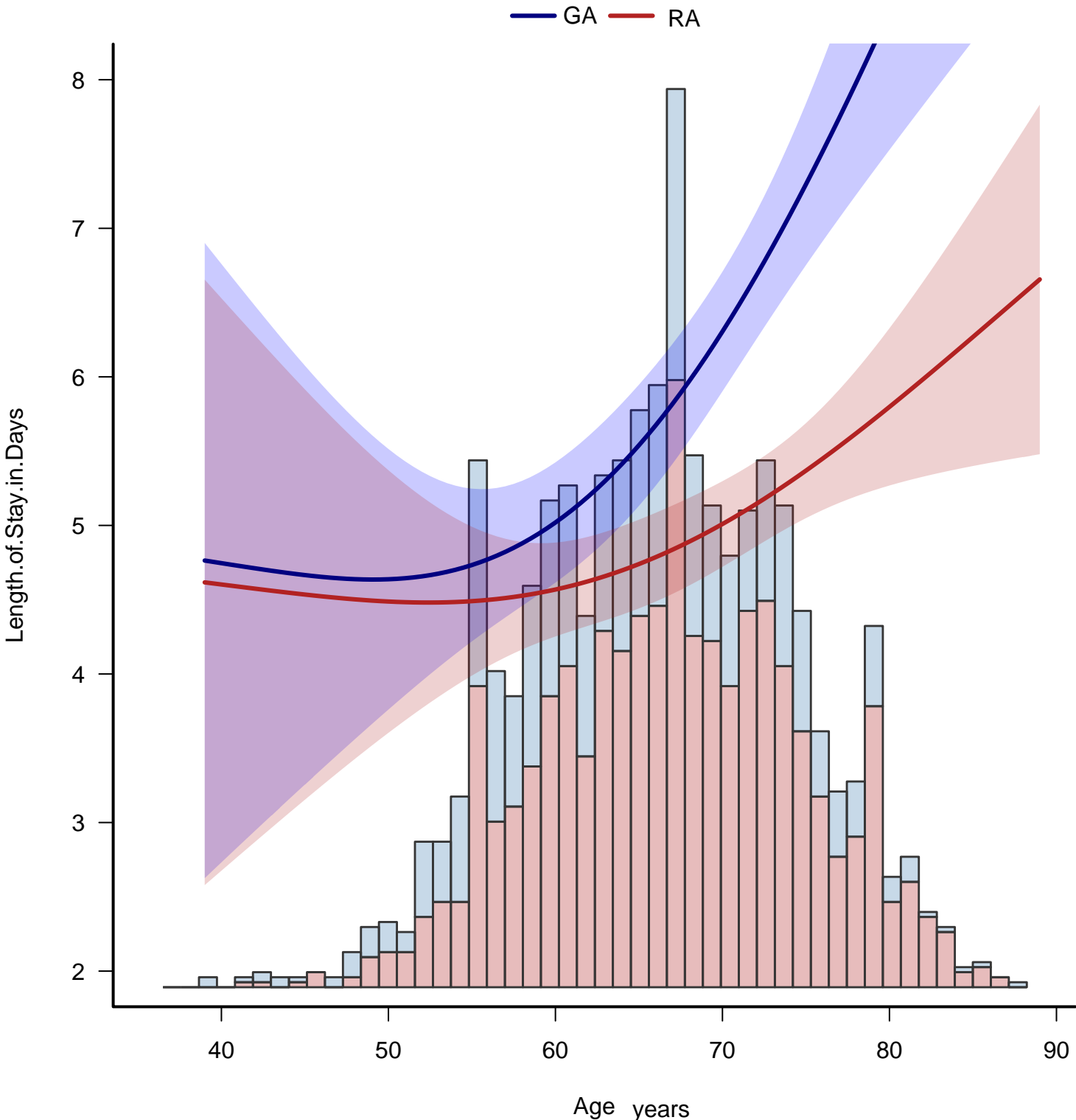

Supplement: Supplementary file 1 — Additional file 1. The association between age and LOS in different anesthesia group. [file 13018_2021_2817_MOESM1_ESM.pdf]

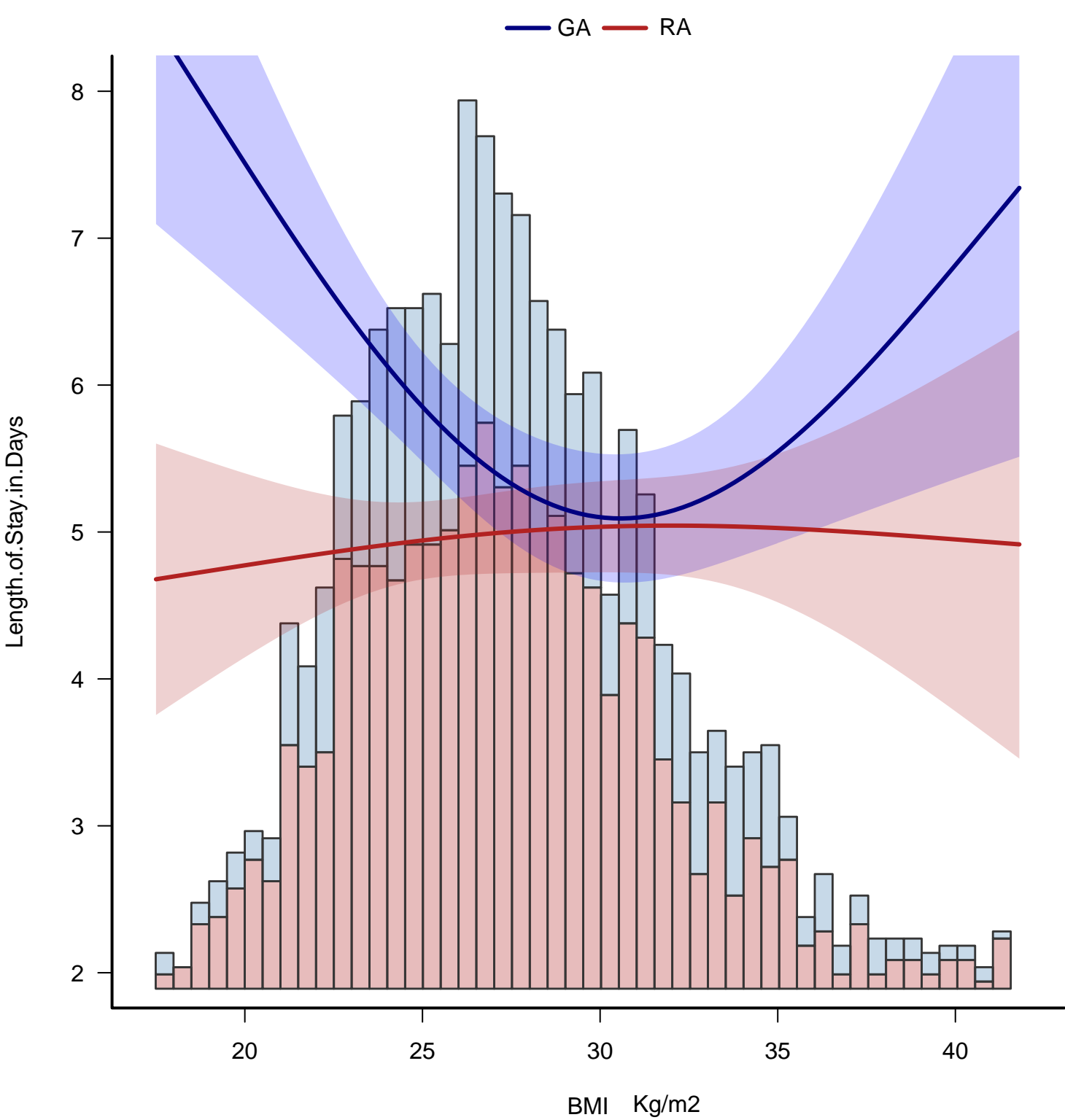

Supplement: Supplementary file 2 — Additional file 2. The association between BMI and LOS in different anesthesia group. [file 13018_2021_2817_MOESM2_ESM.pdf]
